# Dielectricity of a molecularly crowded solution accelerates NTP misincorporation during RNA-dependent RNA polymerization by T7 RNA polymerase

**DOI:** 10.1038/s41598-022-05136-8

**Published:** 2022-01-21

**Authors:** Shuntaro Takahashi, Saki Matsumoto, Pallavi Chilka, Saptarshi Ghosh, Hiromichi Okura, Naoki Sugimoto

**Affiliations:** 1 Frontier Institute for Biomolecular Engineering Research (FIBER), Konan University, 7-1-20 Minatojima-Minamimachi, Kobe, 650-0047 Japan; 2 Graduate School of Frontiers of Innovative Research in Science and Technology (FIRST), Konan University, 7-1-20 Minatojima-Minamimachi, Kobe, 650-0047 Japan

**Keywords:** Nucleic acids, Biochemistry, Physical chemistry

## Abstract

In biological systems, the synthesis of nucleic acids, such as DNA and RNA, is catalyzed by enzymes in various aqueous solutions. However, substrate specificity is derived from the chemical properties of the residues, which implies that perturbations of the solution environment may cause changes in the fidelity of the reaction. Here, we investigated non-promoter-based synthesis of RNA using T7 RNA polymerase (T7 RNAP) directed by an RNA template in the presence of polyethylene glycol (PEG) of various molecular weights, which can affect polymerization fidelity by altering the solution properties. We found that the mismatch extensions of RNA propagated downstream polymerization. Furthermore, PEG promoted the polymerization of non-complementary ribonucleoside triphosphates, mainly due to the decrease in the dielectric constant of the solution. These results indicate that the mismatch extension of RNA-dependent RNA polymerization by T7 RNAP is driven by the stacking interaction of bases of the primer end and the incorporated nucleotide triphosphates (NTP) rather than base pairing between them. Thus, proteinaceous RNA polymerase may display different substrate specificity with changes in dielectricity caused by molecular crowding conditions, which can result in increased genetic diversity without proteinaceous modification.

## Introduction

Biological systems use nucleic acids, such as DNA and RNA, as genetic materials. Nucleic acids are polymers of nucleotides that include genetic information. To obtain and transfer genetic information, the catalysis of polymerization of nucleotides, such as transcription and replication, is a vital process for all life forms. These reactions are carried out in cells by polymerases due to template-dependent nucleotide polymerizations. According to the rules of Watson–Crick base pairing, the polymerizations of complementary nucleotides are catalyzed accurately^1^. However, polymerases sometimes incorrectly incorporate the substrate nucleotides, causing mutations. These mutations can result in a critical deficiency of genetic information. In contrast, from the perspective of species evolution, a low fidelity of polymerization enhances the diversity of genetic sequences^2,3^. As demonstrated by the recent pandemic of the novel severe acute respiratory syndrome coronavirus 2 (SARS-Cov-2), RNA viruses have extremely high rates of spontaneous mutation. It has been suggested that environmental change caused the enhancement of their genetic diversity, which was advantage for survival of viruses and microbes^4^. It has been reported that the in vivo mutation rate in viruses is two orders of magnitude higher than that predicted by in vitro studies^5^. Thus, the effect of the environments of the polymerase reaction on the mismatch incorporation of nucleotide substrates is of interest.

In living cells, DNA and RNA synthesis are catalyzed by various aqueous solutions. The selection of nucleotide substrates depends on the stability of the base pair intermediate. In fact, the solution environments drastically affect the stability of the base pairs. The determinants of stability in nucleic acid duplexes are hydrogen bonding between the bases and base stacking with the nearest-neighbor base pair^6,7^. A high cosolute concentration is associated with molecular crowding, which critically changes the stability of nucleic acids due to decreases in the dielectric constant^8^, decreases in water activity^9–11^, increases in viscosity^12^, and increases in excluded volume^13^. In particular, the Watson–Crick base pairs become unstable, although the edge of the duplex is stabilized under molecular crowding conditions^14–16^. Furthermore, molecular crowding only slightly destabilizes duplex structures having mismatched base pairs compared to that without mismatched pairs^17^. These altered properties can affect forces for substrate recognition by polymerases^18^. Thus, molecular crowding induce mutations in the genome of living systems. Interestingly, the evolution of life is accompanied by molecular crowding. The appearance of the protocell could be attributed to compartmentalization via vesicle formation and coacervate formation^19,20^, where not only organic compounds but also cosolute showed large changes in their concentrations^21,22^. According to phylogenetic analysis, genetic materials were sequestered within membranes when cells became to replicate DNA developed after evolution led to organelle formation^23,24^. Additionally, crowded environments may have facilitated non-enzymatic RNA and DNA replication^25^. Thus, it is possible that the solution environments of polymerase reactions influence genome replication during the evolution of life. Recently, we reported the effect of polyethylene glycol (PEG) as a crowder on the RNA-dependent polymerization of nucleoside triphosphates (NTPs) and 2′-deoxyribonucleoside triphosphates (dNTPs)^26^; PEG with an average molecular weight of 200 (PEG200) reduced the RNA synthesis activity of T7 RNAP and simultaneously enhanced dNTP polymerization. This suggests that the substrate specificity of polymerase was dramatically regulated by the solution environment. The increased misincorporation during RNA polymerization demonstrated that a decrease in the dielectric constant in the solution stabilized the interactions between T7 RNAP and non-complementary ribonucleotides via 2-OH. Because evolutionarily older (precursor) enzymes have broader specificity, the transformability of the bisubstrate specificity of T7 RNAP suggests a similar transient evolutionary process with a role for RNAP in the transition from the RNA–Protein (RNP) world to the DNA world. In addition to the interaction between the 2′OH of NTP and T7 RNAP, the incorporation of the substrates should be discriminated by base pairing with the template strand. Interestingly, there is a compensatory relationship between the energies of hydrogen bonding and those of stacking interactions^27^, which suggests that the misincorporation of NTPs can be facilitated by enhancing the stacking interaction more than hydrogen bonding under certain solution conditions. However, although the effect of PEG on the efficiency and fidelity of the RNA-dependent polymerization by T7 RNAP was investigated^26^, fidelity upon further extension of the replicated polymer has not yet been analyzed.

In this study, we investigated the effects of PEG as a crowder on the mismatch extension of the RNA-dependent polymerization of NTPs by T7 RNAP. Compared with the primer extension from the matched primer end, the reaction from the mismatched primer end indicated that the mismatched NTPs were preferentially incorporated more than the matched NTP. Interestingly, these preferences of incorporated NTPs could be regulated by changes in the solvation properties of NTPs and primer-template RNAs using different cosolutes, such as PEGs with different molecular weights. These results indicate that the degree of decrease in the dielectric constant regulated the incorporation of NTP into the active site of T7 RNAP and base stacking between the NTP and the primer end. This finding suggests that the solvation property of NTP and the primer strand is one of the major factors that facilitates the incorporations of NTP mismatches during polymerase reactions.

## Materials and methods

### Materials

NTP solutions were purchased from Thermo Fisher Scientific (Waltham, MA, USA), and dNTP solutions were purchased from Toyobo (Osaka, Japan). Ethylene glycol (EG), PEG200, and PEG2000 were purchased from Wako Pure Chemicals (Osaka, Japan) and used without further purification. T7 RNAP was purchased from Takara Bio (Shiga, Japan). Other reagents were purchased from Wako Pure Chemicals. FAM-labeled primers and template RNA were purchased from the Japan Bio Service (Saitama, Japan). All RNA sequences used in this study for replication assays are listed in Table 1.

**Table 1:** Sequences of RNA template and RNA primers.

RNAs	Sequences^a,b^
Template	5′-GUCAAUGACACGCUUCGCAC**GGUUGGCAG**AAAAAAAAAA-3′
Primer A	5′-FAM-**CUGCCAACC**A-3′
Primer C	5′-FAM-**CUGCCAACC**C-3′
Primer G	5′-FAM-**CUGCCAACCG**-3′
Primer U	5′-FAM-**CUGCCAACC**U-3′
Complementary strand	5′-C**GGUUGGCAG**-Dabcyl-3′

^a^Bold font indicates complementary regions of template and primers.

^b^Underline of the primers indicates mismatched bases to template sequence.

### Polymerase activity assay and analysis

T7 RNAP (0.5 μM) was incubated with 0.5 μM FAM-labeled RNA primer and 0.5 μM RNA template in 50 mM Tris–HCl (pH 8.0), 10 mM MgCl_2_, 5 mM DTT, the indicated NTPs or dNTPs, and the indicated crowding agent at 25 °C for 12 h (in a total volume of 2.5 μL). After incubation, 5 μL of loading solution, consisting of 3 × loading dye (1.8% Ficoll PM70, 3.0 mM EDTA, and 0.015% bromophenol blue), 125 mM EDTA, 4.8 M urea, and 2.6 ng/μL competing template, was added and heated at 80 °C for 5 min. The products were separated by denaturing polyacrylamide gel electrophoresis (20% acrylamide, 8 M urea in Tris–borate-EDTA buffer) at 70 °C, and the bands were quantified. The percentage of primers extended was calculated as the fluorescence intensity (LAU/mm^2^) of the bands of the extended primers divided by the summed fluorescence intensities (LAU/mm^2^) of all detectable bands. The data for T7 RNAP in the presence of PEG200 are shown as the average of six samples. All other data are shown as the average of the three samples. Errors were represented as standard deviations.

### Fluorescence melting assay

Each FAM-labeled primer was mixed with a Dabcyl-labeled template sequence that contained only the counterpart sequence (5′-GGUUGGCAG-[Dabcyl]-3′) in 50 mM Tris–HCl (pH 8.0), 10 mM MgCl_2_, and 5 mM DTT. The final concentration of the RNA duplexes was 200 nM. The RNA-containing solution was heated at 95 °C and then cooled to 25 °C at a rate of − 1 °C/min. The fluorescence was measured using Mx3000P (Agilent Technology) at 0.5 °C at a heating rate of 0.5 °C/min.

### Measurements of solution properties

Dielectric constant was measured by using the fluorescent probe 1,8-ANS (1-anilino-8-naphthalene sulfonate) in sample solutions containing EG, PEG200, or PEG2000 at 25 °C. The fluorescence emission spectrum of the probe shows a blue shift in the response to changes in media of low dielectric constant. The spectra excited at 356 nm were taken using a fluorescence spectrophotometer (JASCO, F-6500). The dielectric constant was calculated using a standard curve of several organic solvents with known values.

## Results

### Incorporation of matched NTP next to the mismatched primer end

**Figure 1:**
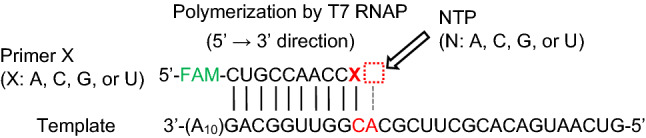
Sequences of template and primer RNAs used in this study. X indicates each 3′ terminal base of primers. The dashed square on the right side of “X” of the primer end indicates the position of incorporated NTP. Four different primers and four NTPs were used. As a primer end, primer G is the matched one complementary to the C base of the template. As an incorporated NTP, UTP is the matched one complementary to the A base of the template.

We chose T7 RNAP as a model polymerase to compare the mismatch extensions of both RNA and DNA polymerizations, as previously reported^26^. The RNA template and primers used in this study are listed in Table 1. The length of the RNA template was 39 nucleotides (nt), which included a 9–10-nt primer-complementary sequence (Fig. 1). One of the primers, named primer G, contains a 10-nt full-match template-complementary sequence, whereas the other primers, namely, A, C, and U, each contain a mismatched base at the 3 terminus. After polymerase reaction, the products were analyzed by denaturing polyacrylamide gel electrophoresis. The fractions of the extended primers were estimated from the decreased intensities of the initial primer bands.

**Figure 2:**
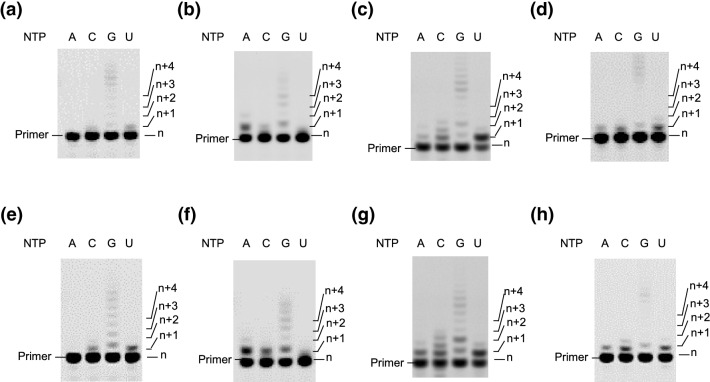
Polymerization in the absence of PEG200 by T7 RNAP with (**a**) primer A, (**b**) primer C, (**c**) primer G, (**d**) primer U, and in the presence of PEG200 with (**e**) primer A, (**f**) primer C, (**g**) primer G, (**h**) primer U. Positions of products (*n* + 1, 2, 3, or 4, where *n* is the primer length) are indicated. Conditions: 0.5 μM T7 RNAP, 0.5 μM each RNA primer, 0.5 μM template RNA with 100 μM each NTP in 50 mM Tris–HCl (pH 8.0), 10 mM MgCl_2_, and with or without 20wt% PEG200 at 25 °C for 12 h. The results using primer G are the original data of PAGE, which was not shown in the previous report^26^. Full-length gels are presented in Supplementary Information Figure S7.

To analyze the efficiencies of mismatch polymerization with each NTP at the first position next to the 3′ terminus of each primer, we incubated each primer and each NTP in the absence and presence of PEG200 (Fig. 2). Table 2 shows a summary of the extended primers with NTPs at the first position next to the 3′ terminus of each primer in the absence and presence of PEG200. In the absence of PEG200, the extensions of UTP, which were the complementary nucleotides to the position to be incorporated, showed that the terminal mismatch using primers A, C, and U incorporated small UTP incorporation (8.4%, 12%, and 16% of each primer extended, respectively) at the 3′ terminal end compared with that in the case of primer G having matched primer end (50%)^26^. This indicates that matched extension from the mismatched 3′-end of the primer inefficiently occurred for the RNA primer extension by T7 RNAP. Among these primers for 3 mismatches, the efficiency of UTP incorporation was higher for primers U, C, and A, in that order. These results suggest that the geometry of the terminal mismatch base pairing affected the subsequent UTP incorporation to catalyze the polymerization. In the duplex structure, each strand interacts with anti-parallel polarity, and the C1′-C1′ distance of Watson–Crick base pairs in a *cis* orientation is maintained as 10.3 ± 0.2 Å^28^. Because some polymerases strictly recognize their substrates based on size^29^, T7 RNAP may distinguish the size of the template-complementary substrate. Based on the identified RNA base pairs in a *cis* orientation, some possible base pairs might form in each primer case (Fig. S1)^30^. Primer A can form a C·A (C of template and A of primer A) mismatch, while no mismatch base pair has been reported where C base in a *cis* Watson–Crick orientation, except for the case where A is protonated under acidic conditions, presenting a C1-C1 distance of 10.4 Å. For primer C, the mismatch base pairing could form in an orientation of *cis* Watson–Crick and Hoogsteen base pair with 9.9 Å of a C1-C1 distance. In the case of Primer U, the C·U Watson–Crick base pair can also form with the same orientation as the C·C mismatch base pair with 10.5 Å a C1-C1 distance. Thus, the 3′ terminus of primer U may be at an appropriate C1-C1 distance to form matched Watson–Crick base pairs more than that of primer C. Primer A did not form a base pair well under neutral pH. In addition, the 3′terminal ends of primer C may be located differently from the terminal ends of the matched primer, resulting in the inefficient incorporation of NTPs forming a Watson–Crick base pair with the base of the template in the next position.

**Table 2:** Percentages of extended primers with NTPs at the first position next to the 3′ terminal of each primer in 0 or 20 wt% PEG200.

Substrate	Primer							
	Primer A		Primer C		Primer G (Matched)		Primer U	
	(-)PEG200	( +)PEG200	(−)PEG200	( +)PEG200	(−)PEG200	( +)PEG200	(−)PEG200	( +)PEG200
ATP	0^a^	0^a^	30 ± 2.2	39 ± 1.4	9.0 ± 2.2^b^	25 ± 8.1^b^	8.8 ± 0.6	13 ± 1.7
CTP	7.5 ± 1.2	18 ± 1.7	15 ± 1.7	30 ± 0.6	29 ± 11^b^	35 ± 13^b^	13 ± 0.9	25 ± 1.2
GTP	21 ± 7.0	28 ± 5.0	33 ± 5.2	56 ± 10	23 ± 12^b^	33 ± 20^b^	18 ± 1.1	34 ± 7.1
UTP (Matched)	8.4 ± 1.4	17 ± 3.4	12 ± 0.7	14 ± 1.5	50 ± 14^b^	37 ± 20^b^	16 ± 4.1	17 ± 4.1

^a^No bands for extended primers were detectable.

^b^Data were taken from the reference^26^.

### Stability of template-primer duplex in the absence of PEG200

**Figure 3:**
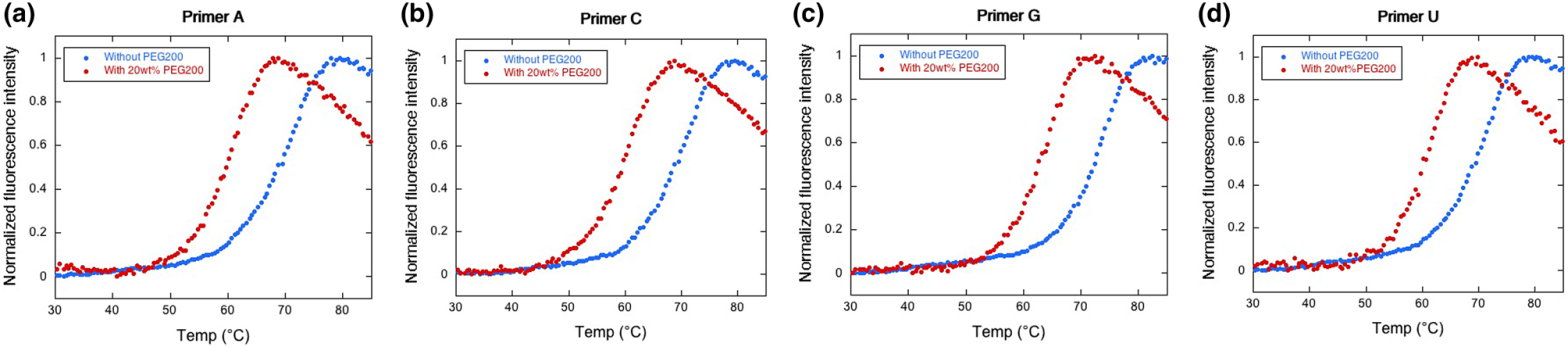
Fluorescence melting of the RNA duplexes composed with template strand and (**a**) primer A, (**b**) primer C, (**c**) primer G, and (**d**) primer U, respectively. The measurements were carried out in 200 nM each RNA duplex, 50 mM Tris–HCl (pH 8.0), 10 mM MgCl_2_, and 5 mM DTT.

To evaluate the interaction between the primer end and the template RNAs, we analyzed the thermal stability of the template-primer duplexes in the absence of PEG200. It was revealed that the selection of NTP in the active site of T7 RNAP requires triple interactions (base pairing from the template nucleotide, base stacking from the 3′ RNA terminal nucleotide, and hydrophobic contacts from residues M635 and Y639) to act together to position the NTP base in a catalytically competent conformation^31^. The last hydrophobic contacts can act as a cover to stabilize the NTP in the active site of the polymerase. Therefore, this study also suggests that the selection of NTP depends on base pairing and stacking interactions with template strands, and the interaction with the protein can be used as a constraint for the correct position of the NTP. Our experimental setup for the melting assay included these three points (base pairing, base stacking, and a constraint of the position by connecting to the primer strand with the phosphate backbone) and can be used as a model system for the selection of NTPs in the polymerase. As a counterpart of the fluorescence-labeled primer strands, the quencher-labeled complementary sequence (complementary strand shown in Table 1) with a mismatch at the primer end was used. All duplexes showed a sigmoidal increase in fluorescence with increasing temperature, indicating temperature-dependent melting of the duplexes (Fig. 3). As shown in Table 3, the melting temperature (*T*_m_) of the duplex with primer G was the highest (73.5 °C), with primer strands that fully matched the template strands in a Watson–Crick base pairing manner. On the other hand, other primers decreased the *T*_m_ values, and the differences were very subtle (A: 71.0 °C, C: 70.4 °C, and U: 71.5 °C). It has been reported that a mismatch base pair at the duplex end can be formed and stabilize the duplex, which suggests that the mismatch base pairs on these duplexes were formed^32^. Thus, the preference for the incorporation of the NTPs may depend on the geometry of the structure of the 3′-end of the primer facing the active center of T7 RNAP.

**Table 3:** Melting temperature of primer and template RNA.

	Primer A	Primer C	Primer G (Matched)	Primer U
*T*_m_ (°C) in the absence of PEG200	71.0 ± 0.1	70.4 ± 0.1	73.5 ± 0.2	71.5 ± 0.1
*T*_m_ (°C) in the presence of 20wt% PEG200	62.5 ± 0.3	62.5 ± 0.2	65.4 ± 0.1	63.0 ± 0.4

### Incorporation of mismatched NTPs next to the mismatched primer end

Next, we checked the primer extensions with mismatched NTPs, including ATP, CTP, and GTP. Although the matched primer (primer G) preferred the matched NTP (UTP), other mismatch extensions from mismatched primers emerged in the absence of PEG200 (Fig. 2 and Table 2). Most of the results indicated the elongation of the primers. In the case of mismatch extensions with GTP, the ladder product should be a slippage product, which is commonly observed in the reaction of T7 RNAP in the presence of only GTP^33,34^. To reduce the slippage reaction, we ran the polymerization of GTP with a template containing A, G, or U at the counter position of the primer end (Fig. S2). However, all results showed a ladder of products, which suggests that the polymerization of GTP likely occurred because of the thermodynamic stability of the mismatch base pairing. The yields of G-ladder in the case of primers G and A were larger than those of other primers, indicating that the stacking between the primer end and GTP was important. Therefore, the first incorporation of GTP occurred at position A on the template. Interestingly, the percentages of extended primers with mismatched GTP were higher than those with matched UTP with each primer. The highest level of mismatch extension was observed for primers C and GTP (33%). The value was approximately threefold higher than that of the same primer C with template-complementary UTP. Among the three mismatched primers, the efficiencies of mismatch extensions with GTP decreased in the following order: primer C (33%) > primer A (21%) > primer U (18%). These efficiencies were comparable to those of the matched primer G (23%). Thus, the consecutive incorporation of mismatched GTP occurred in the first mismatch as well as in the matched end. The second highest magnitude of the incorporation of mismatch NTP was for primer C with ATP (30%). Notably, the top two efficiencies were observed for the purine NTPs. The efficiency of ATP incorporation with primer U (8.8%) was relatively lower than that with UTP, and the extension of primer A did not occur, which may indicate that ATP could not bind to the catalytic center of T7 RNAP because of the steric hindrance of A·A mismatch (incorporated ATP and A base of template). In the case of extension with CTP, the magnitude of the extensions (primer A, 7.5%; primer C, 15%; and primer U, 13%) were similar to those observed in the case of UTP (Table 2). These results indicate that the trend of incorporation of NTPs depended on the base structure, whether it was purine or pyrimidine. The preference for purine GTP suggests that incorporation was accelerated by base stacking. However, the incorporation of ATP was unfavorable in each case. This may be due to the steric geometry of C·A (C of template and A of primer A) mismatch and the next A base of the template and incorporation of ATP. For the formation of *cis* Watson–Crick conformations involving the mismatches, A·A (A of template and A of incorporating ATP) and A·G (A of template and G of incorporating GTP) mismatches require C1-C1 distances of 12.3 and 12.5 Å, respectively, while A·G can also form base pairs in a *cis* Watson–Crick and Hoogsteen orientation with a C1-C1 distance of 10.5 Å (Fig. S3). An A·C (A of template and C of incorporating CTP) mismatch requires a C1′-C1′ distance longer than 10.4 Å to form a *cis* Watson–Crick conformation, as described above. Thus, mismatch extension with ATP and GTP from primers C and U occurs due to the similar lengths of the expanded C1-C1 distances of the terminal mismatches and C1-C1 distances of newly formed bonds to form the *cis* Watson–Crick conformation in the diluted solution. Thus, T7 RNAP is prone to incorporate an NTP driven by base stacking with the primer end, but is repressed by steric geometry at the terminal bases next to the mismatched terminal.

### Incorporation of mismatched NTPs next to the mismatched primer end in the presence of PEG200

To determine the effect of the solution on the mismatched primer extension, we measured the T7 RNAP reaction in the presence of 20 wt% PEG200 (Fig. 2e-h). In the solution containing PEG200, as we reported previously, the efficiency of the extension of the primer with both matched primer and substrate (primer G and UTP) decreased (50% to 37%) (Table 2)^26^; however, PEG200 did not change the incorporation of UTP with primer C (12% to 14%) and primer U (16% to 17%), except for an increase in efficiency with primer A (8.4% to 17%). For mismatched NTP incorporation in the presence of 20 wt% PEG200, all the efficiencies with any of the primers increased compared with those in the absence of PEG200 (Table 2). To examine the acceleration of NTP incorporation for the mismatched primers, we checked the stability of the primer-template duplexes in the presence of 20 wt% PEG200 (Fig. 3 and Table 3). Fluorescence melting indicated that the *T*_m_ values decreased with the addition of 20 wt% PEG200 in each case. As observed in the absence of PEG200, the matched primer G showed the highest *T*_m_ value, whereas other mismatched primers showed lower values. The decrease in ∆*T*_m_ from those in the absence of PEG200 were − 8.5 °C for primer A, − 7.9 °C for primer C, and − 8.5 °C for primer U. We proposed that PEG200 destabilizes the duplex of nucleic acids via Watson–Crick base pairing by decreasing the water activity^35^. Compared with the ∆*T*_m_ value of primer G (− 8.1 °C), the magnitudes of the reduction of mismatched primers were comparable. Given that the destabilization effect of PEG200 is due to the formation of base-pairing, the stability in the presence of PEG200 should decrease with an increase in the magnitude of interactions by base pairings^6^. These results suggest that each primer end formed a different type of base pairing with the template, which resulted in destabilization by PEG200 via different manners.

### Effect of physicochemical properties on the mismatch extension

**Figure 4:**
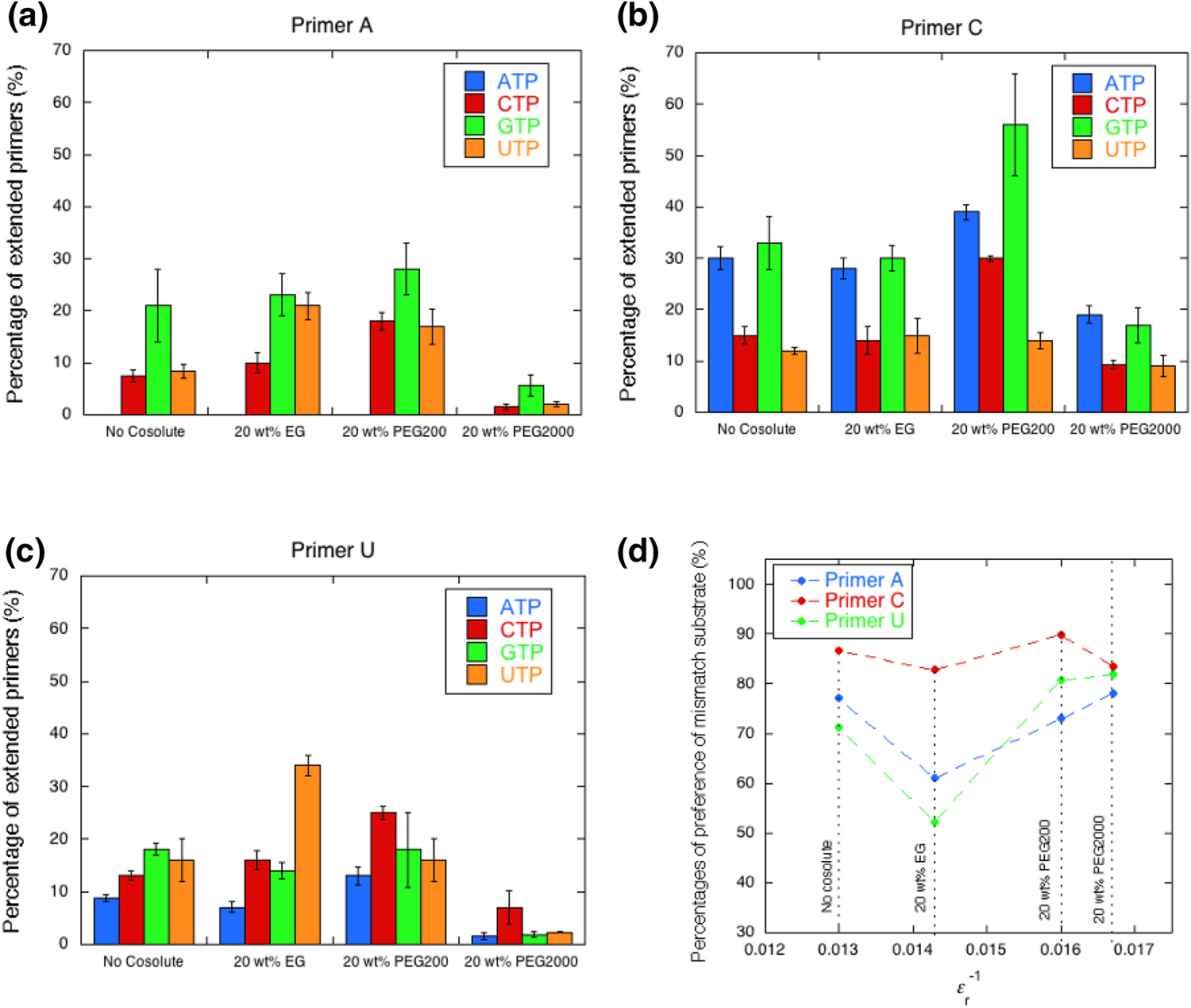
Percentages of extended primers with NTPs in each solution at the first position next to the 3′ terminal of (**a**) primer A, (**b**) primer C, and (**c**) primer U. (**d**) Percentage of the preference of mismatched substrate (sum of percentage of extended primer (%) with ATP, CTP, and GTP) over the mismatch primer extension (sum of percentage of extended primer (%) with all NTPs).

To further investigate the effect of the solution on mismatch primer extension, we used PEG with different molecular weights. Ethylene glycol (EG) and PEG2000 were used in this study. Due to the difference in the PEG structure, the physicochemical properties, which in turn change the solvation properties, such as water activity and dielectric constant (*ε*_r_), can be differentially changed^36^. Among these conditions, the solution containing 20 wt% EG showed the largest dielectric constant (*ε*_r_ = 70.0), while that containing 20 wt% PEG200 showed the middle (*ε*_r_ = 62.5), and 20 wt% PEG2000 had the lowest dielectric constant (*ε*_r_ = 54.6) at 25 °C. We carried out primer extensions in the presence of the same concentration (20 wt%) of PEG (Fig. S4 and Table 4). Figures 4a–c show the percentages of extension from each mismatch primer with NTPs in each solution. The relatively low yields of the primer extensions under 20wt% PEG2000 condition were due to the instability of T7 RNAP (Fig. S5). However, the polymerase reactions in this study were performed at 25 °C (not 37 °C), which should minimize concerns about the effect of the stability of the polymerase in this study. As shown in these figures, there were two types of trends in terms of the amount of primer extensions in each condition: with primers A and U (Fig. 4a, c) and with primer C (Fig. 4b). In the case of primers A and U, the results indicate that the incorporation of the matched UTP was highest in the solution containing 20 wt% EG (Fig. 4a, c). This implies that lowering the dielectric constant facilitated the electrostatic interaction between the substrate and the active site of T7 RNAP, which accelerated the incorporation of the matched substrate. Furthermore, as EG showed the largest decrease in water activity among the PEGs tested here, there are other possibilities for the UTP incorporation. One is the facilitation of the hydrolysis reaction of the primer extension, since the equilibrium of the hydrolysis of the NTP in the polymerase reaction is generally followed by a decrease in water activity. Second, the incorporation of matched NTPs could require less hydration than that of mismatched NTPs. However, the melting assays in the presence of PEG indicated that the ∆*T*_m_ values of RNA duplexes with terminal mismatch compared with the values in the absence and presence of 20 wt% PEG200 (cases of primers A: ∆*T*_m_ = 8.5 °C, primer C: ∆*T*_m_ = 7.9 °C, and primer U: ∆*T*_m_ = 8.5 °C) was almost the same as that of the full-match duplex (∆*T*_m_ = 8.1 °C) (Table 3), which suggests that the water activity did not significantly affect the mismatch incorporation. Furthermore, a low level of UTP incorporation was observed under the 20 wt% PEG200 and PEG2000 conditions compared with the incorporation of mismatched NTPs (Fig. 4d). Based on the RNA primer extension along the DNA template, we recently found that the primer extension with matched NTP was reduced in solutions with more than 0.016 of *ε*_r_^−1^^36^. However, with mismatched NTPs, the highest levels in the condition were over 0.016 of *ε*_r_^−1^, before reducing with a further large *ε*_r_^−1^. This preference for NTPs suggests that the mismatch extension was also accelerated by base stacking. The potential energy of the interactions between induced dipole moments, which is the main stabilization factor in base stacking^37,38^, and the electric static interactions have a ratio of the inverse square of *ε*_r_. We found that the primer extensions decreased in all cases with each NTP as a substrate with increasing KCl concentration (Fig. S6). The dielectric constant in the solution containing 100 mM KCl in the absence of cosolute (*ε*_r_ = 76.3) at 25 °C was approximately equal to that of the solution containing 1.0 wt% PEG200 (*ε*_r_ = 73.9). These results indicate that NTP incorporation was mainly driven via electrostatic interaction, which includes not only the interaction between NTP and the active site of the protein but also the stacking interaction caused by dipole moments and/or induced dipole moments. The relatively high incorporation of purine NTPs (ATP and GTP) at the lower concentration of KCl might indicate that the contribution of the stacking interaction via purine bases was relatively higher than that of pyrimidine NTPs. Therefore, interactions between induced dipole moments could cause the substrate-dependent effects of solution properties on primer extension with a preference of mismatched NTPs over matched ones.

**Table 4:** Percentages of extended primers with NTPs at the first position next to the 3′ terminal of each primer in 20 wt% EG and PEG2000.

Substrate	Primer					
	Primer A		Primer C		Primer U	
	EG	PEG2000	EG	PEG2000	EG	PEG2000
ATP	0^a^	0^a^	28 ± 2.1	19 ± 1.7	7.1 ± 1.1	1.6 ± 0.6
CTP	10 ± 1.9	1.5 ± 0.6	14 ± 2.8	9.3 ± 0.8	16 ± 1.8	7.0 ± 3.2
GTP	23 ± 4.0	5.6 ± 2.0	30 ± 2.5	17 ± 3.4	14 ± 1.6	1.9 ± 0.5
UTP (Matched)	21 ± 2.6	2.0 ± 0.5	15 ± 3.4	9.0 ± 2.0	34 ± 2.0	2.3 ± 0.1

^a^No bands for extended primers were detectable.

With regard to the excluded volume effect, the volumetric effect of incorporating NTP stacked at the end of the primer should be considered. In the case of DNA, it has been reported that the order of volumetric contribution to the formation of the nearest neighbor base pair was TG < TC < TA < TT^39^. This order indicates how the excluded volume effect destabilizes base stacking. Conversely, the order of TT < TA < TC < TG is the expected order for substrate selection of the primer extension because the reaction with smaller volumetric changes is stabilized by the excluded volume effect. The primer extension from primer U in the presence of 20wt% PEG2000, which is the largest cosolute in this study, dominantly reacted with CTP, which is different from that in the absence of PEG2000. Considering the expected order by the excluded volume effect, UC is more favorably incorporated than UU and UA. Therefore, the excluded volume effect was not only substantial for substrate selection during primer extension but might also play a role in incorporation of NTPs.

For primer C, the preference of mismatched NTPs for primer extension was not greatly altered (Fig. 4b and 4d). This result implies that primer C had no preference for NTPs, as discussed in the results in the absence of PEGs. Thus, T7 RNAP incorporated NTPs via base stacking more than Watson–Crick base pairings. In fact, T7 RNAP preferred purine NTPs for primer C because the purine bases have strong stacking interactions. These results suggest that the solution environment regulates matched or mismatched primer extensions based on the interaction between the primer ends.

## Discussion

As solvent selection is vital for the catalytic reaction in organic chemistry, solvation around the catalyst plays a significant role in determining the efficiency of the reaction. Water is the main solvent found in nature, with enzymes catalyzing various reactions in water. Therefore, solvation (hydration) around the substrate and/or enzymes is likely to be the key factor driving reactions. For nucleic acids, solvation around the nucleic acid structures plays a crucial role in their stability, and any change in the solvation layer (e.g. changes in the concentration of ions or cosolutes in the medium) results in alterations in the stability and reactivity of nucleic acids. Several studies have been conducted on the role of solvation and psychochemical properties, such as water activity and medium dielectricity, under crowding conditions on the different types of nucleic acid structures^40–43^. However, much less is known about the effect of these properties on the biological reactions involving nucleic acids. For reactions such as replication and transcription, the major focus is on the specificity of the enzyme towards the substrate or the chemical interaction between nucleic acids and polymerases. Akabayov et al. studied the effect of macromolecular crowding on the replication of T7 bacteriophage based on physical properties, such as volume exclusion and conformation of the polymerase^44^. However, considering the polyanionic nature of nucleic acids and presence of charged amino acid residues in polymerase, physicochemical properties such as the polarity of the medium should have an effect on the course and outcome of reactions.

Our results indicate that the solution environment changed with PEG, promoting NTP misincorporation beyond Watson–Crick base pairing due to alterations in the dielectricity of the medium. A decrease in the dielectric constant of the medium upon the addition of cosolutes was found to promote NTP misincorporation in two ways: by increasing the stacking interaction between the existing and incorporated NTPs, and by overcoming the substrate specificity due to screening of the ionic interaction between the NTPs and charged amino acid residues (Lys and Arg) at the catalytic center of T7 RNAP^45^. The ionic interaction between the negatively charged NTPs and positively charged amino acid residues resulted in a conformational change of RNAP to orient NTPs at the Mg^2+^ binding site for polymerization. An enhancement in the ionic interaction between NTPs and amino acids under low dielectric medium was found to potentially promote the direct incorporation of NTPs at the substrate-binding site, governed by the enhanced stacking interaction between NTP and the terminal base of the primer. These findings suggest that molecular crowding induced changes in solvation properties, resulting in NTP misincorporation, which may lead to genetic diversity.

Previously, we reported that T7 RNAP preferentially matched dNTP to rNTP for primer extension in the presence of high concentrations of PEG200, indicating that RNA polymerases play a role in polymerizing DNA, triggered by molecular crowding during the transition from RNA–protein (RNP) to DNA^26^. In contrast, the results obtained in the present study suggest that it is the molecular environment that mainly decreases the dielectric constant to promote mismatch extension of the template non-complementary NTPs once misincorporation has occurred. This misincorporation causes mutations in replication and transcription, thereby increasing the diversity of genetic information during evolution. In contrast, PEG conditions maintained the efficiencies of the extensions of template non-complementary dNTPs at lower levels than those of template complementary dNTPs^26^. Thus, alterations in the dielectricity of the medium may have induced the formation of diversified genetic information in early evolution, before the emergence of DNA, as well as promoting the fixation of genetic information on DNA in the process of transfer of genetic material from RNA to DNA. These findings may be related to evolutionary processes. Furthermore, phylogenetic analysis revealed that an evolutionary common ancestor, which originated from the genomes of the phage, plasmid, and nucleolus, contained T7-like single-subunit RNAPs, and that a DNA polymerase or reverse transcriptase of the modern world evolved from the ancestral T7-like single-subunit RNAPs^46^. Thus, the predicted effects of the solution environment on genetic evolution likely occurred in the probiotic world. Furthermore, as T7 RNAP is an enzyme of the current era, the modulation of substrate specificity may commonly occur in the life cycle of RNA viruses, including SARS-Cov-2. The in vivo mutation rate of viruses is known to be two orders of magnitude higher than that predicted by in vitro studies^5^. As shown in the case of RNA-dependent RNA polymerase in RNA viruses, the fidelity of RNA polymerization has been investigated by using structural analyses such as X-ray crystallography, molecular dynamics simulations, and NMR spectroscopy^47,48^. To determine the mechanism of the misincorporation of NTPs, it is important to pursue the detailed structure of the active center of the enzyme in the crowding condition. Thus, molecular crowding has enormous potential for regulating the substrate selectivity of virus RNAP by changing the solvation properties of nucleic acids without causing protein modification.

## Supplementary Information

Supplementary Information.
