# Supplementary material for: Dielectricity of a molecularly crowded solution accelerates NTP misincorporation during RNA-dependent RNA polymerization by T7 RNA polymerase
Source: Sci Rep. 2022 Jan 21;12:1149. doi: 10.1038/s41598-022-05136-8 (PMC8782835; doi:10.1038/s41598-022-05136-8)
Supplement: Supplementary file 1 — Supplementary Information. [file 41598_2022_5136_MOESM1_ESM.pdf]

Dielectricity of a molecularly crowded solution accelerates NTP misincorporation during RNA-dependent RNA polymerization by T7 RNA polymerase

Shuntaro Takahashi[1,a], Saki Matsumoto[1,a], Pallavi Chilka[1], Saptarshi Ghosh[1], Hiromichi Okura[1], and Naoki Sugimoto[1,2*]

[1]Frontier Institute for Biomolecular Engineering Research (FIBER), Konan University, 7-1-20 Minatojima-Minamimachi, Kobe 650-0047, Japan.

[2]Graduate School of Frontiers of Innovative Research in Science and Technology (FIRST), Konan University, 7-1-20 Minatojima-Minamimachi, Kobe 650-0047, Japan.

* To whom correspondence should be addressed:

Tel.: (+81)78-303-1457

Fax: (+81)78-303-1495

Email: sugimoto@konan-u.ac.jp

[a] The authors contributed equally to this work.

CONTENTS

Figure S1. Potential base pairings at the end of duplexes with each primer and template RNA

Figure S2. The primer extension with a template without C in the presence of only GTP

Figure S3. Potential base pairings between template RNA (A base) and each incorporated mismatched NTP

Figure S4. Polymerization by T7 RNAP in the presence of 20wt% EG

Figure S5. CD melting of T7 RNAP with or without cosolute

Figure S6. Effect of KCl concentration on primer extension in the absence of PEG

Figure S7. Full-length gels images of Figure 2

Figure S8. Full-length gels images of Figure S2

Figure S9. Full-length gels images of Figure S4

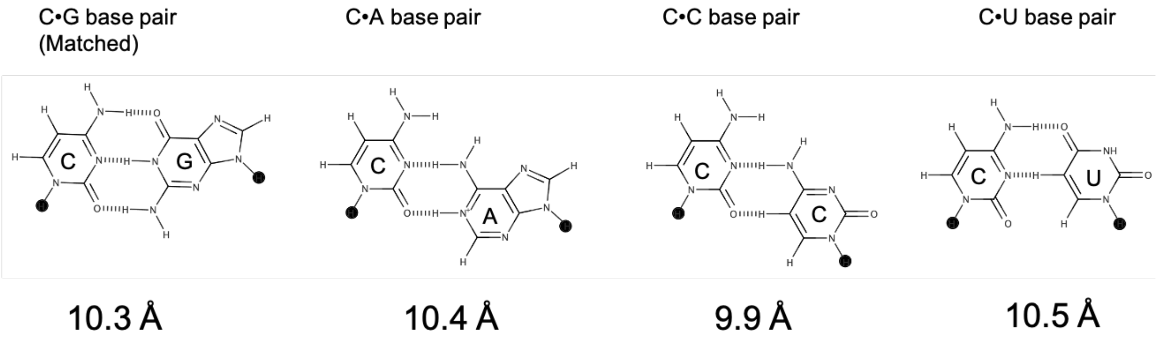

Figure S1. Potential base pairings at the end of duplexes with each primer and template RNA. The C1’-C1’ distances are shown below the structures. Black circles indicate the C1’ position. The data were obtained from the reference S1.

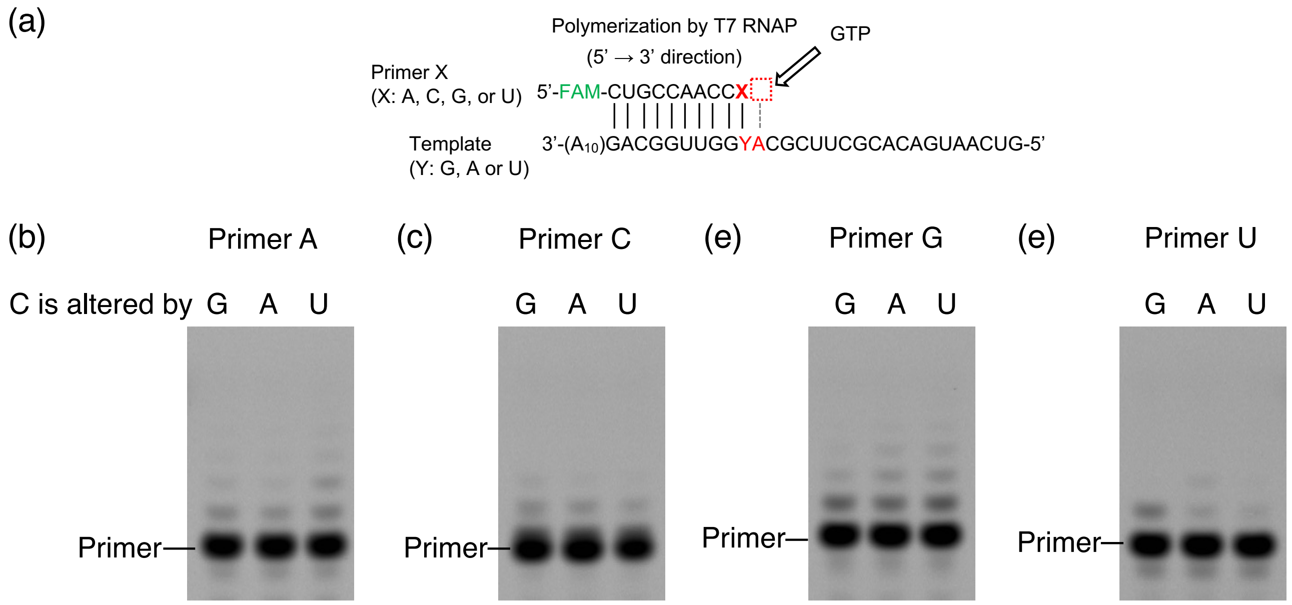

Figure S2. The primer extension with a template having G, A, or U at the counter position of the primer end in the presence of only GTP. (a) The experimental setup. The position "Y" on the template is G, A, or U. Incorporation of GTP by T7 RNAP with (b) primer A, (c) primer C, (d) primer G, (e) primer U and templates in which C is altered by G, A, or U. Conditions: 0.5 μM T7 RNAP, 0.5 μM each RNA primer, 0.5 μM template RNA with 100 μM of each NTP in 50 mM Tris-HCl (pH 8.0), 10 mM $MgCl_2$ at 25°C for 12 h. Full-length gels are presented in Supplementary Information Figure S8.

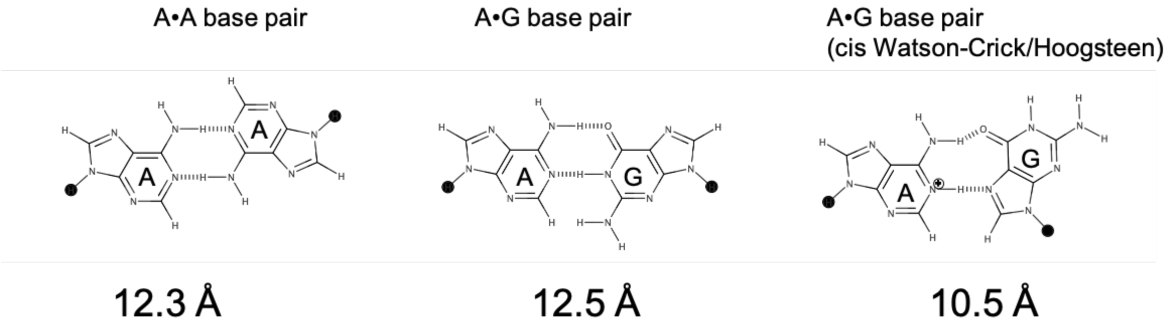

Figure S3. Potential base pairings between template RNA (A base) and each incorporated mismatched NTP. The C1'-C1' distances are shown below the structures. Black circles indicate the C1' position. The data were obtained from the reference S1.

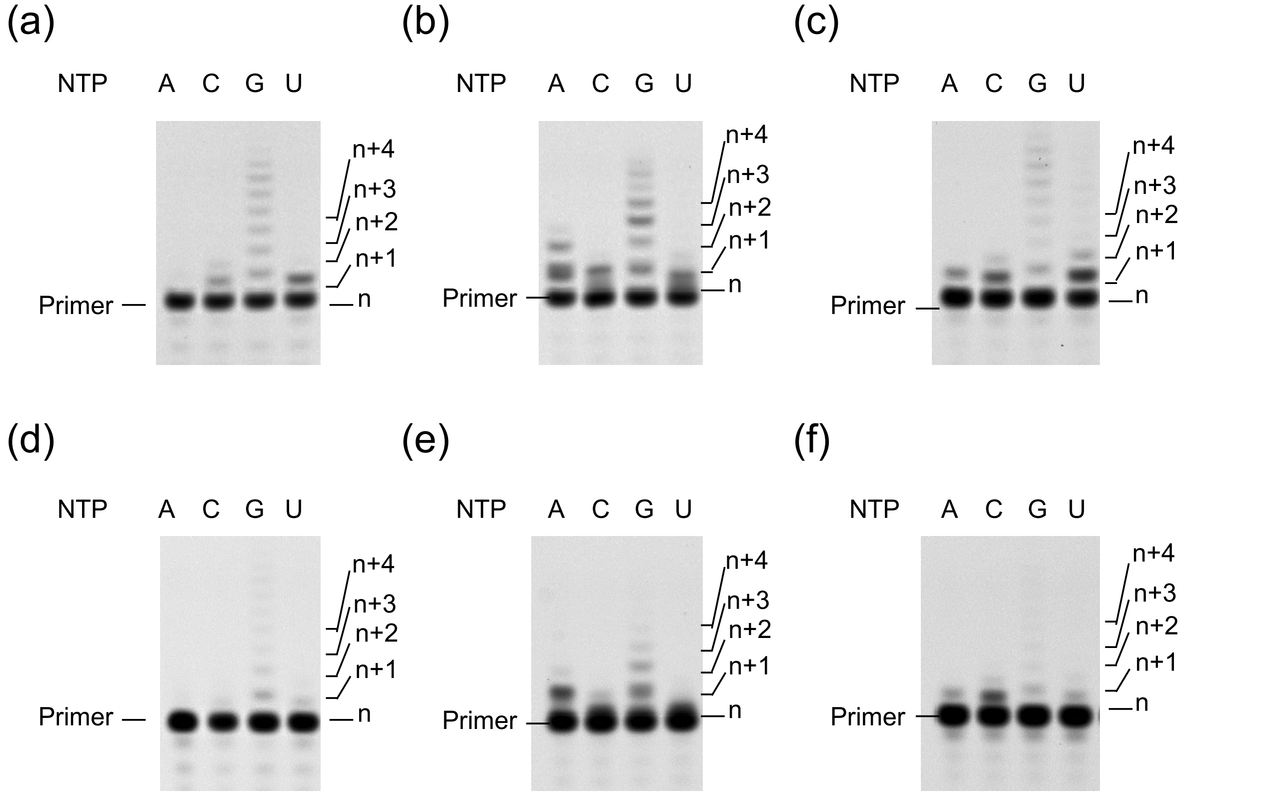

Figure S4. Polymerization by T7 RNAP in the presence of 20wt% EG with (a) primer A, (b) primer C, and (c) primer U, and in the presence of 20wt% PEG2000 with (d) primer A, (e) primer C, and (f) primer U. The positions of products (*n*+1, 2, 3, or 4, where *n* is the primer length) are indicated. Conditions: 0.5 μM T7 RNAP, 0.5 μM each RNA primer, 0.5 μM template RNA with 100 μM each NTP in 50 mM Tris-HCl (pH 8.0), 10 mM $MgCl_2$, and 20wt% PEG200 at 25°C for 12 h. Full-length gels are presented in Supplementary Information Figure S9.

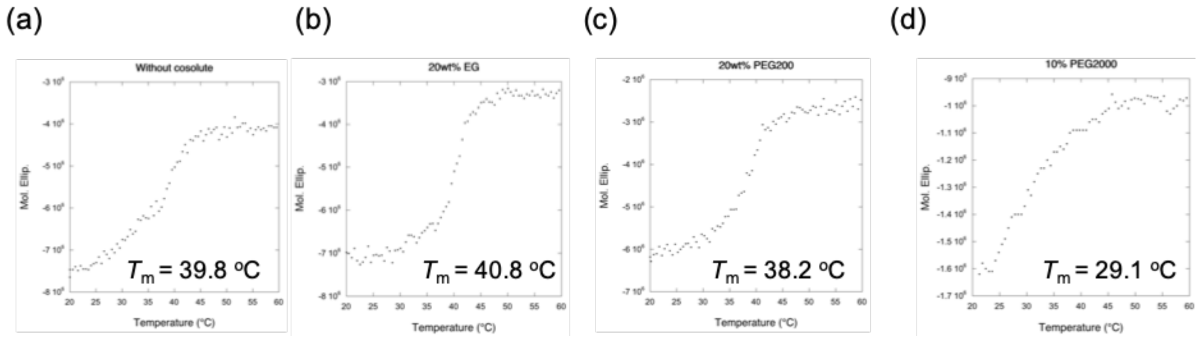

Figure S5. CD melting of T7 RNAP with or without cosolute. The CD signals were tracked at 222 nm. Conditions: 0.5 μM T7 RNAP, in 50 mM Tris-HCl (pH 8.0), 10 mM $MgCl_2$, in the absence (a) or presence of 20wt% EG (b), 20wt% PEG200 (c), or 10wt% PEG2000 (d). Due to the instability of T7 RNAP in the presence of 20wt% PEG2000, 10wt% PEG2000 was used.

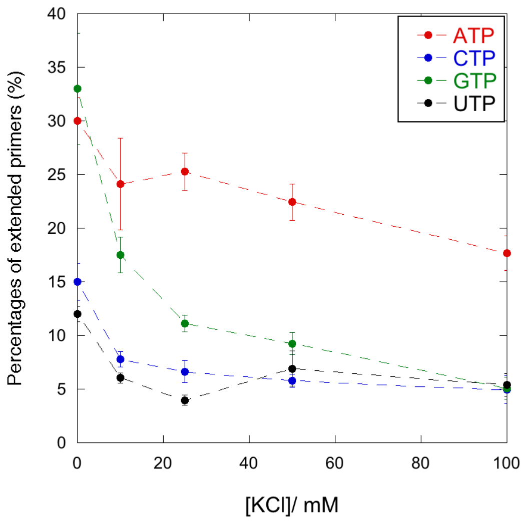

Figure S6. Effect of KCl concentration on primer extension in the absence of PEG. Primers extended with ATP, CTP, GTP, and UTP are indicated in red, blue, green, and black, respectively. The data points associated with 0, 10, 25, 50 and 100 mM KCl deviated from the linear plots. All samples were incubated in 50 mM Tris-HCl (pH 8.0), 10 mM $MgCl_2$ at 25 °C for 12 h.

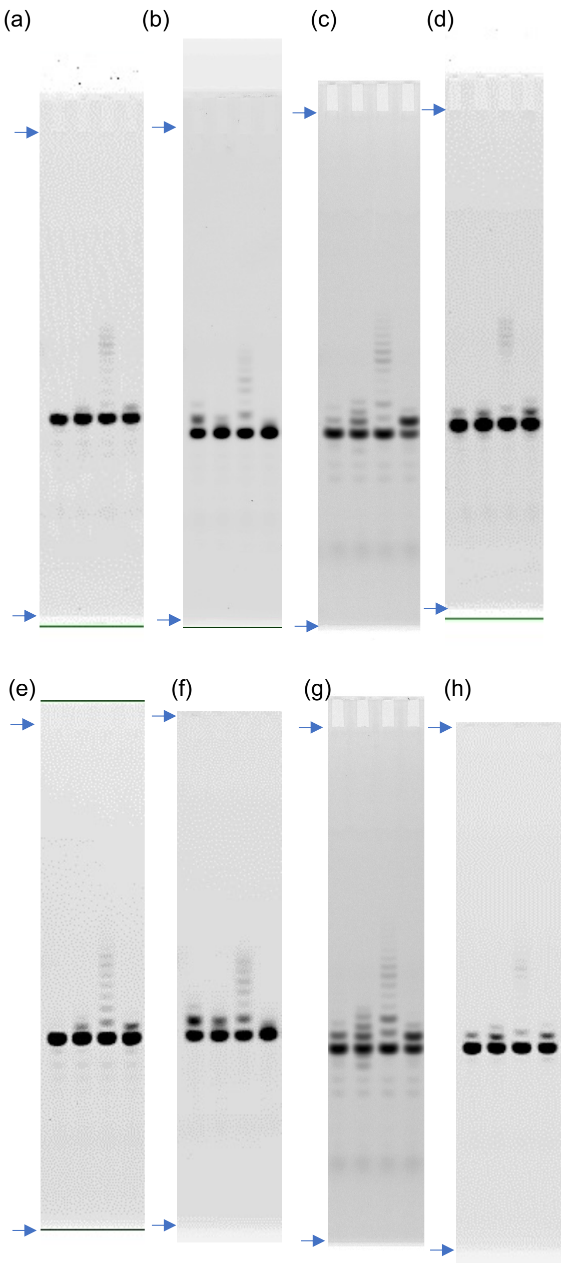

Figure S7. Full-length gels images of Figure 2 indicating polymerization in the absence of PEG200 by T7 RNAP with (a) primer A, (b) primer C, (c) primer G, (d) primer U, and in the presence of PEG200 with (e) primer A, (f) primer C, (g) primer G, (h) primer U. The arrows show the edges of the gels.

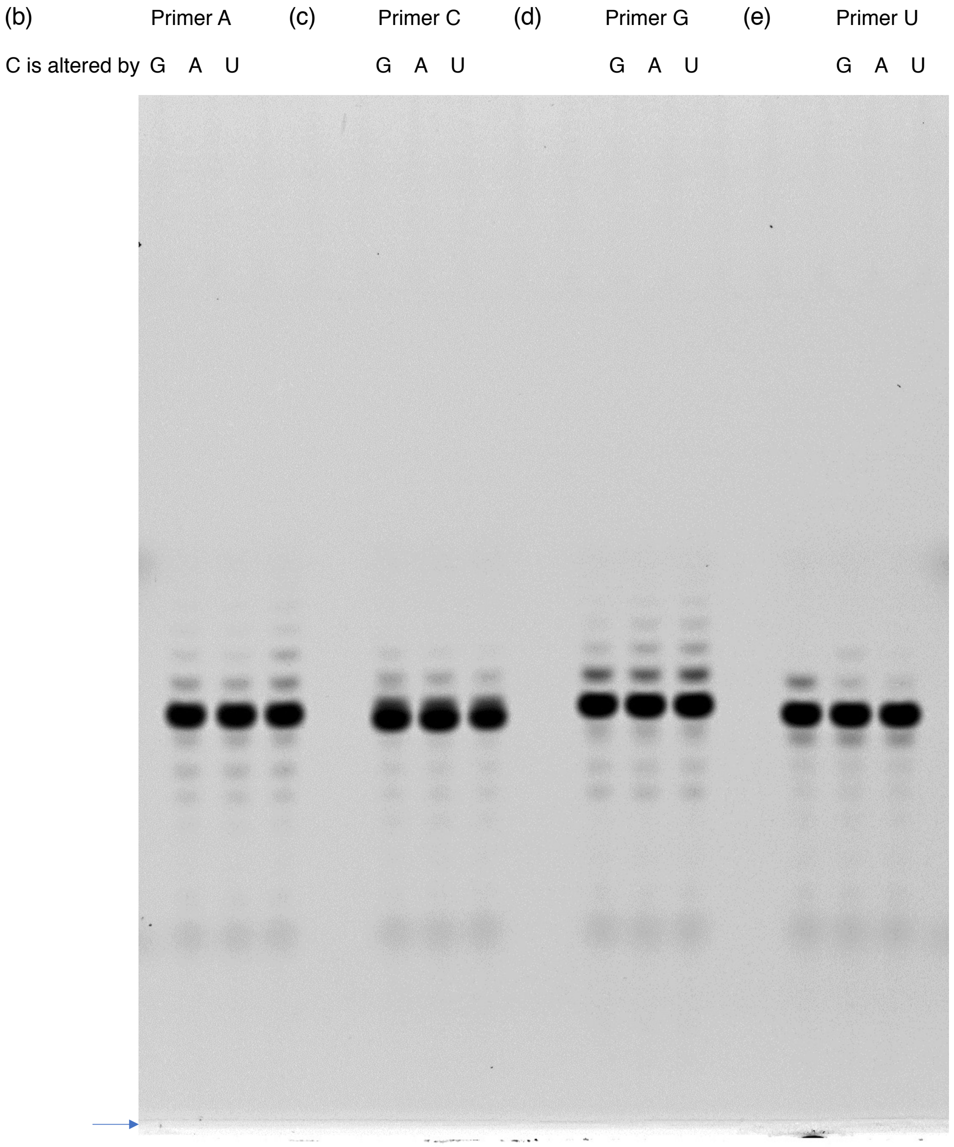

Figure S8. Full-length gels images of Figure S2 indicating the primer extension with a template having G, A, or U at the counter position of the primer end in the presence of only GTP. (a) The experimental setup. The position “Y” on the template is G, A, or U. Incorporation of GTP by T7 RNAP with (b) primer A, (c) primer C, (d) primer G, (e) primer U and templates in which C is altered by G, A, or U. The arrow shows the bottom edge of the gels. The top edge of the gel was not photographed in the experiment.

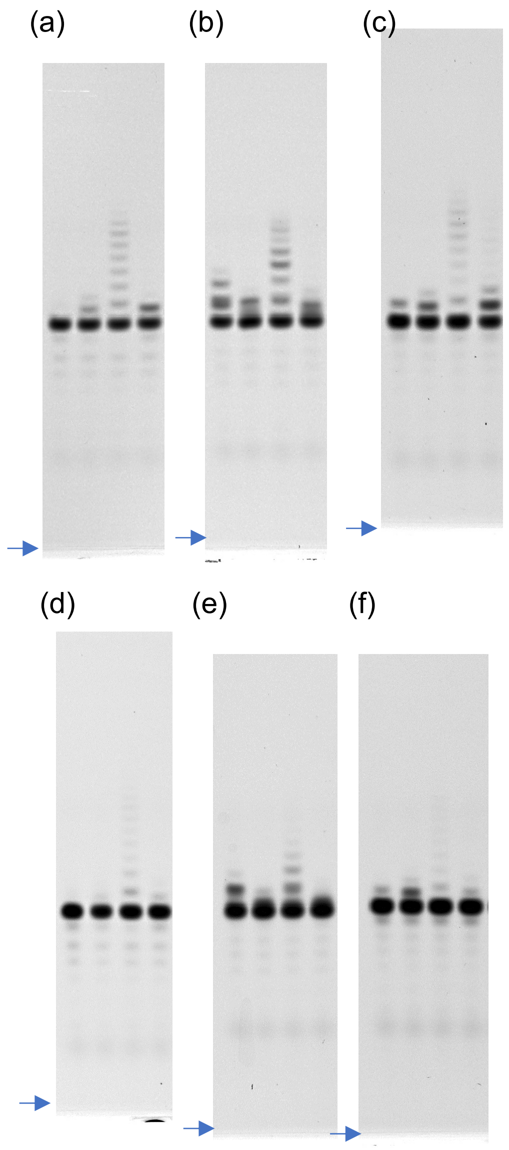

Figure S9. Full-length gels images of Figure S4 indicating polymerization by T7 RNAP in the presence of 20wt% EG with (a) primer A, (b) primer C, and (c) primer U, and in the presence of 20wt% PEG2000 with (d) primer A, (e) primer C, and (f) primer U. The arrows show the bottom edges of the gels. The top edges of the gels were not photographed in the experiments.

Reference

S1 Leontis, N. B.; Stombaugh, J.; Westhof, E. *Nucleic Acids Res.* 2002, *30*, 3497.
